# Database analysis of children and adolescents with Bipolar Disorder consuming a micronutrient formula

**DOI:** 10.1186/1471-244X-10-74

**Published:** 2010-09-28

**Authors:** Julia J Rucklidge, Dermot Gately, Bonnie J Kaplan

**Affiliations:** 1 Department of Psychology, University of Canterbury, Christchurch, New Zealand; 2 Department of Economics, New York University, New York, USA; 3 Department of Pediatrics, Community Health Sciences, University of Calgary, Calgary, Canada

## Abstract

**Background:**

Eleven previous reports have shown potential benefit of a 36-ingredient micronutrient formula (known as EMPowerplus) for the treatment of psychiatric symptoms. The current study asked whether children (7-18 years) with pediatric bipolar disorder (PBD) benefited from this same micronutrient formula; the impact of Attention-Deficit/Hyperactivity Disorder (ADHD) on their response was also evaluated.

**Methods:**

Data were available from an existing database for 120 children whose parents reported a diagnosis of PBD; 79% were taking psychiatric medications that are used to treat mood disorders; 24% were also reported as ADHD. Using Last Observation Carried Forward (LOCF), data were analyzed from 3 to 6 months of micronutrient use.

**Results:**

At LOCF, mean symptom severity of bipolar symptoms was 46% lower than baseline (effect size (ES) = 0.78) (*p *< 0.001). In terms of responder status, 46% experienced >50% improvement at LOCF, with 38% still taking psychiatric medication (52% drop from baseline) but at much lower levels (74% reduction in number of medications being used from baseline). The results were similar for those with both ADHD and PBD: a 43% decline in PBD symptoms (ES = 0.72) and 40% in ADHD symptoms (ES = 0.62). An alternative sample of children with just ADHD symptoms (n = 41) showed a 47% reduction in symptoms from baseline to LOCF (ES = 1.04). The duration of reductions in symptom severity suggests that benefits were not attributable to placebo/expectancy effects. Similar findings were found for younger and older children and for both sexes.

**Conclusions:**

The data are limited by the open label nature of the study, the lack of a control group, and the inherent self-selection bias. While these data cannot establish efficacy, the results are consistent with a growing body of research suggesting that micronutrients appear to have therapeutic benefit for children with PBD with or without ADHD in the absence of significant side effects and may allow for a reduction in psychiatric medications while improving symptoms. The consistent reporting of positive changes across multiple sites and countries are substantial enough to warrant a call for randomized clinical trials using micronutrients.

## Background

The diagnosis of pediatric bipolar disorder (PBD) is one of the most controversial in modern child psychiatry [1]. Disagreement exists on how to define it, at what age to identify it, and how it matches with the more traditional diagnosis of bipolar disorder in adulthood (see [2] for an extensive review). However, regardless of how it is conceptualized and whether changes in criteria have been validated or supported by research, a consequence of the loosening of the Diagnostic and Statistical Manual's (DSM) definition [3] is that thousands of children and young people have now been diagnosed with PBD and prescribed psychiatric medications that have limited empirical support and that often carry worrisome adverse effects. The changes in the definition of PBD have raised substantial debate [4]. The current DSM-V task group is attempting to address the surge in diagnoses of PBD with the introduction of a new category, Temper Dysregulation Disorder with Dysphoria, which is also controversial [5].

Although there has been a significant increase in the number of studies describing the treatment of PBD over the last decade, there are surprisingly few controlled studies of pharmacotherapy for mania in children and adolescents [6]. Mood stabilizers (e.g., lithium, carbamazepine, valproate acid) have been shown to be less effective in the treatment of PBD (response rate averaging 40%) as compared with adults (response rate averaging 65%) [7]. For example, an open trial of 42 individuals with PBD showed a response rate of 34% for carbamazepine, 42% for lithium and 46% for sodium divalproex [8]. A randomized controlled trial (RCT) comparing lithium to placebo in 25 bipolar adolescents with substance dependency showed a 46.2% response rate [9] compared with 8.3% in the placebo group. A recent double-blind RCT comparing divalproex extended release with placebo in the treatment of PBD in 150 youths found no difference between groups and an overall response rate of 24% [10]; the authors concluded that there was no support for the use of divalproex in the treatment of PBD. However, others have found higher response rates to divalproex in open trials [11]. Based on a comparative analysis of acute randomized placebo controlled trials, Correll et al. [12] found a pooled effect size of .24 (based on change on the Young Mania Rating Scale (YMRS)) for mood stabilizers as compared to placebo in the treatment of acute mania in youth.

Atypical antipsychotics (e.g., aripiprazole, olanzapine, quetiapine, risperidone, ziprasidone) have been increasingly reported to be used as frontline agents for the treatment of PBD and they may be more effective than mood stabilizers in the treatment of acute mania in youth. A recent comparative analysis of acute randomized placebo controlled trials for the treatment of mania in pediatric populations found five trials that had used antipsychotics and estimated a pooled effect size for the YMRS at .65 [12] with all five studies showing efficacy. To the best of our knowledge, all other trials on these medications are either open-label or chart reviews [13,14] and are difficult to summarize given the variation in age, diagnostic methods, outcome measures used, inclusion criteria, and definition of a response. A review of the literature showed that response rates to antipsychotic treatments for PBD can vary from 38% to 80% [15]. Although antipsychotics may be more effective than the mood stabilizers, they may also carry a higher risk profile in terms of adverse effects [12].

One group of children who have proved to be particularly difficult to manage is those who are diagnosed with both Attention-Deficit/Hyperactivity Disorder (ADHD) and PBD. The rate of ADHD comorbidity in pediatric bipolar populations ranges from 57% to 93% [16-18]. There is very little empirical data to support the use of specific medications for these young people [7,19]. Indeed, ADHD has been found to be one predictor of treatment nonresponse in PBD with mixed or manic episodes [20,21] suggesting that this combination of disorders is linked to poorer outcomes. Effects of medications are modest in those with both disorders, with responses to mood stabilizers in small open label trials ranging from 20-29% [13,22].

In contrast, medications typically used to treat ADHD have been used with this population with better success, at least in controlling the ADHD symptoms in PBD children and adolescents. Hah and Chang [23] reported on 7 patients with both ADHD and PBD who were treated with atomoxetine in conjunction with mood stabilizers. They found that 6 of the 7 improved in symptoms of ADHD and none of the patients had a manic or hypomanic episode during the treatment period which ranged from 1.5 months to 18 months. Sheffer et al. [11] studied a sample of 30 bipolar patients with ADHD (6-17 years) in a 4-week placebo-controlled trial using mixed amphetamine salts after being stabilized with divalproex sodium using an 8 week open-label trial. Although ADHD symptoms were not improved by divalproex sodium, the RCT phase showed amphetamine salts resulted in 89.6% of the sample receiving a Clinical Global Impression (CGI) rating of much or very much improved compared with 10% on the placebo.

Although there are a range of psychopharmacological options available to try to treat emerging mood symptoms in children and adolescents, the results to date indicate that many are not responding to these treatments [20], especially when co-occurring disorders are included in samples. The limited response in combination with the adverse effects associated with these medications (such as adiposity, cardiac changes, neuromuscular effects, hypokinesias, and hyperandrogenism [6]), some of which may be more substantial for youth as compared with adults [12], indicate the need to investigate other treatment options for them.

The literature on nonpharmacological biological treatments for PBD is sparse. The trials on omega-3 fatty acids have been mixed, with as many showing no effect as showing an effect (see [24] for a review). There are virtually no good trials on the use of St. John's wort, kava kava, or S-adenosyl-L-methionine (SAMe) for the treatment of PBD. There is, however, a growing body of literature reporting that a multi-ingredient approach (such as combinations of minerals and vitamins) can be a successful alternative for the treatment of unstable mood, in both children and adults.

As recently reviewed [25], 100 years of scientific research of single-nutrient interventions has provided some promising (though modest) results. In contrast, research since 2000 on multinutrient formulas has shown much larger effects on mood. An older version of the 36-ingredient formula (EMPowerplus) evaluated in the current set of analyses has been studied in a variety of ways (the ingredients of the formula are listed on the developer's website (Truehope.com) and in Additional File 1: it consists of 14 vitamins, 16 minerals, 3 amino acids, and 3 antioxidants). There have been four publications of open-label trials in adults, adolescents, and children with bipolar disorder [26-29]. In addition, two children with mood swings and explosive rage were studied in within-subject cross-over designs: on-off control of their tantrums and rages was demonstrated with this same formula [30]. Further, in a database analysis of a large sample of 358 adults with bipolar disorder, more than half were positive responders (defined as >50% decrease in symptom severity) after 3 months of consuming this micronutrient formula [31]. Importantly, their symptom improvement was sustained at 6 months, making it unlikely that placebo or expectancy effects accounted for the reported changes.

Using a newer formulation but the same ingredients (to reduce the number of capsules, the processing method changed in November 2002, resulting in a decrease from 32 to 15 capsules per day for the full adult dose), five additional reports have been published. A case report of an 18-year-old boy with obsessive-compulsive disorder (OCD) was published with historical data showing his response to cognitive behavior therapy [32]. Subsequently, he was treated with the 36-ingredient formula in an ABAB design, which resulted in on-off control of anxiety and mood symptoms [32]. Another case has been reported of a child diagnosed with bipolar disorder and with six years of well-documented pharmaceutical treatments for his psychiatric symptomatology [33]. The child and his family chose to transition from medication onto the micronutrient formula when he was 12, resulting in a resolution of all psychiatric symptoms. An open-label trial with 14 adults with ADHD and mood dysregulation showed significant improvement in both ADHD and mood symptoms over an 8 week period with a 2 month follow up showing maintained changes in those who chose to stay on the formula [34]. One case from this trial was observed over a one year period and showed off-on-off-on control of symptoms when she stopped and started the formula [35]. Finally, a case control study of 44 children and youth with autism spectrum disorder (ASD) whose mood and irritability symptoms were treated with the same formula; they were matched by age, sex, and socio-economic status with 44 individuals who were treated with conventional medications [36]. Although both groups improved significantly, those treated with the micronutrient formula improved much more, especially in terms of mood and irritability, and they reported only about one-sixth as many adverse events, and no weight gain. Several other studies of this same formula are under review and in progress. While there are no completed RCTs on this formula, there have been numerous RCTs on micronutrients in general showing efficacy in the treatment of violent behavior in incarcerated populations [37,38], slowing cognitive decline in individuals with dementia [39], and reducing behavioral problems in school populations [40]. There is greater variability in results when studies use fewer ingredients: for example, an RCT using a nonclinical and primarily non-depressed sample of older men did not show improved depression after two years consuming three (B_12_, B_6 _and folic acid) of the 36 micronutrients used in the current study [41].

In addition to reporting positive results in various types of patients studied with a variety of experimental designs, all the findings on adverse events are of particular importance for children: the only adverse events reported have been the occasional minor stomach ache or headache, but there have been no reports of the more serious adverse events commonly found with pharmaceuticals (constipation, dry mouth, dyskinesia, tachycardia, akathisia, etc.).

In summary, while some conventional treatments of PBD are showing efficacy in reducing the acute symptoms of bipolar disorder, they do carry with them a high risk of significant side effects warranting the need for investigations into other viable treatments. There are now 11 consistently positive reports conducted independently from the manufacturer across different sites and countries showing amelioration of unstable mood and anxiety in children and adults following treatment with a micronutrient formula. We report here a new set of database analyses, similar to the one published by Gately and Kaplan [31] in adults. For the current report, the focus is on PBD.

## Methods

### Data source

The analysis is based on the data provided by people who purchased a micronutrient formula (called EMPowerplus) and provided checklist data on their symptom severity to the product's developers. The formula contains primarily vitamins and minerals, and most people find it while searching the internet to learn about natural treatments of mental disorders. An unusual characteristic of the way in which the company sells this formula enabled the authors to perform the analyses described here: the company has a telephone support line, consisting of people who keep in touch with clients to educate them about the use of the product, and to track problems and successes, which can be done either by phone, fax or internet. People who want to buy the formula only for general health can just purchase it by phone and it is mailed to them. But people who want to take this formula for amelioration of psychiatric or neurologic symptoms are encouraged to use a checklist to monitor their progress, using symptoms primarily derived from the DSM-IV [42]. The Self-Monitoring Form which forms the basis of the current analyses consists of 16 DSM-specified mood symptoms (e.g., loss of interest in hobbies or activities; an excessively high or elated mood). Clients or their parents rated each symptom from 0 (not at all) to 3 (very much), for a maximum score of 48. Use of the Self-Monitoring Form is voluntary, so not everyone chooses to use it. The database used in these analyses was anonymous, using assigned identifier numbers.

A similar scale is used for the symptoms of ADHD: 8 symptoms are scored from 0 - 3. However, the symptoms listed for the ADHD scale include mood symptoms. Consequently, we chose to report on only the first three items of the scale as those items were specific to ADHD (inattention, impulsivity and hyperactivity). See Additional file 2 Table S2 for the specific items for both scales. This database analysis received ethics approval from the Conjoint Health Research Ethics Board at the University of Calgary, Faculty of Medicine.

### Subjects and Materials

Data were available from clients who provided information to the company's database from January 2001 (when the database was incorporated into standard use by the company) through August 2007 about their children's behavior. There were 709 children in the database aged from 7 years to less than 18 years at the start of their monitoring whose parents reported that they had been diagnosed with bipolar disorder. However, the majority of those families stopped submitting data within two weeks, making it insufficient time to experience or report therapeutic benefit. Therefore, criteria were established analogous to the previous report on adult clients [31] to strengthen confidence in the reliability of the information being analyzed. The final sample was selected based on the following:

Symptom monitoring. Completing daily symptom checklists and submitting them to the internet or by phone or FAX is a burden for families struggling with these problems, and even the most well-organized, compliant families might not provide daily reports for very long. A minimum requirement was set as the presence of symptom reports for at least 60 of the first 180 days after starting the micronutrients; this minimum resulted in 120 clients (71 males and 49 females) in the Primary Sample (mean age = 12.8, SD = 3.2). This minimum requirement ensured that we were reporting on a group of clients who had likely consumed the product for long enough in order for us to establish the effectiveness of the micronutrients for those clients.

**Figure 1:**
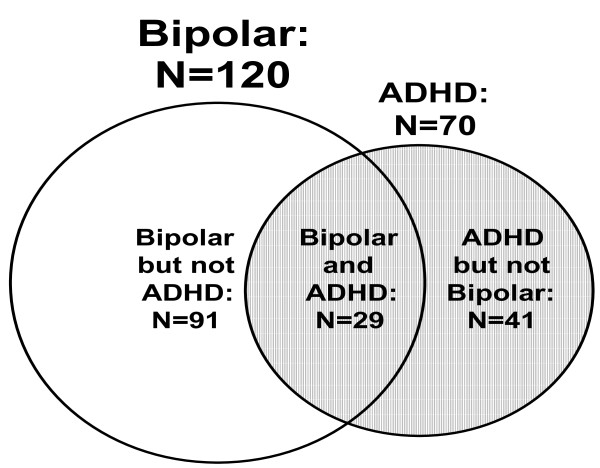
**Venn diagram of client diagnostic groups**.

Diagnoses. Of the 120 clients whose parents had reported them to have been diagnosed with PBD, 29 were reported to have been diagnosed with both ADHD and PBD; the remaining 91 with PBD but not ADHD. In order to be able to compare the change in ADHD symptoms with a group of children without bipolar disorder, we ascertained an additional 41 clients from the database who were identified by their parents as ADHD but not bipolar and who, consistent with the Primary Sample, met the minimum reporting standard on the self-monitoring form (see Figure 1 for a summary of the samples). These clients were taken from a pool of 321 children aged from 7 years to less than 18 years at the start of their monitoring whose parents reported that they had been diagnosed with ADHD but not bipolar disorder.

Although physician confirmation of diagnosis was not available, 79% (n = 95) of the Primary Sample were taking psychiatric medications at the time they commenced taking the micronutrients, indicating that a physician considered their mood and/or attention symptoms to be sufficiently severe to warrant medication. The 20 most-frequently-used medications are listed in Table 1; the distribution of medication use over the course of the study period is in Table 2.

**Table 1:** 20 most commonly prescribed medications taken by the 120 bipolar clients

PRESCRIBED MEDICATIONS	# CLIENTS
**ANTIDEPRESSANTS**	
Serotonin and/or Norepinephrine Reuptake Inhibitors	
Lexapro (escitalopram)	10
Zoloft (sertraline)	8
Strattera (atomoxetine)	6
Prozac (fluxoxetine)	6
Wellbutrin (bupropion)	4
Effexor (venlafaxine)	4
Paxil (paroxetine)	6
Luvox (fluvoxamine)	3
	
**MOOD STABILIZERS**	
Lithium	29
Anticonvulsants	
Depakote (divalproex)	22
Lamictal (lamotrigine)	9
Tripleptal (oxcarbazepine)	6
Tegratol (carbamazepine)	4
Topomax (topiramate)	4
	
**ANTIPSYCHOTICS**	
Risperdal (risperidone)	26
Abilify (aripiprazole)	17
Zyprexa (olanzapine)	8
Geodon (ziprasidone)	8
	
**ANXIOLYTICS**	
Buspar (buspirone)	3
Klonopin (clonazepam)	3

**Table 2:** Psychiatric medication indices

	Medication Index		
		
	Baseline	Last Observation Carried Forward	% change from Baseline
**1. Primary Sample: Bipolar = sum of sub-samples #2 and #3 (N = 120)**			
% taking medication	79%	38%	-52%
Average medication index for those taking medication at Baseline	2.06	0.54	-74%
Average medication index for all clients	1.63	0.43	-74%
			
**2. Sub-Sample: Bipolar but not ADHD (N = 91)**			
% taking medication	80%	38%	-52%
Average medication index for those taking medication at Baseline	2.08	0.52	-75%
Average medication index for all clients	1.67	0.41	-75%
			
**3. Sub-Sample: Bipolar and ADHD (N = 29)**			
% taking medication	76%	38%	-50%
Average medication index for those taking medication at Baseline	2.00	0.60	-70%
Average Medication index for all clients	1.52	0.46	-70%
			
**4. Alternative Sample: ADHD but not Bipolar (N = 41)**			
% taking medication	41%	12%	-71%
Average medication index for those taking medication at Baseline	1.49	0.22	-85%
Average medication index for all clients	0.62	0.09	-85%

Baseline symptom data. The baseline symptom severity index was created from a minimum of three days of symptom data. Most people begin at a very low dose of this formula and titrate upward over the course of several days or a week, which means that when a day or two of data assigned to baseline coincided with the beginning of treatment; symptom changes in the first three days would probably not be attributable to the nutrients. In any case, inclusion of days after treatment onset is a conservative approach which would make it more difficult to show symptom reduction associated with micronutrient use. For those individuals with more than three days of symptom data in the database that preceded the onset of treatment, the baseline index was averaged over all such days.

### Calculation of Last Observation Carried Forward (LOCF)

In order to minimize the potential confound of a placebo effect, we chose to analyze the data up to 6 months post baseline. Forty-nine percent (n = 59) of the Primary Sample reported symptoms through 6 months, 16% (n = 19) through 5 months, 17% (n = 20) through 4 months and 18% (n = 22) through 3 months. As our measure of a client's change in symptom severity, we compared their baseline measure with their Last Observation Carried Forward: averaged over month 6 for 49% of clients, average for month 5 for 16% of clients, and so forth.

### Calculation of Medication Index

There were 25 children who reported consuming no psychiatric medication in the baseline period or during the subsequent 6 months, and 95 who did report medication use for at least part of that time. The Medication Index for each client was calculated in the following manner: a) first, the number of medications was added together; then, b) at any given time point, the Index reflects the dosage in relation to the maximum which that individual consumed. As an example, a client taking four medications in the baseline period at their personal maximum dosage would have a baseline Medication Index of 4, but if the dose of one of the four medications was decreased by 25% in their final reporting month, then their LOCF Medication Index would be 3.75 (cf. Table 2). Mean daily dose of the micronutrient formula at LOCF was 13.7 capsules (SD = 4.8). Due to the documented potentiating effect of micronutrients on medications [43], the micronutrients and medications are cross tapered typically with the assistance of the prescribing physician. In other words, as the micronutrients are introduced medications are systematically reduced.

Database and statistical software used included Access, Excel, and Tableau. Paired t-tests were used to calculate change from baseline to LOCF and Effect Size (ES) calculations were based on Cohen's *d*, calculated as the difference in mean symptom severity at baseline and at LOCF, divided by the standard deviation across clients of the differences between baseline and LOCF.

## Results

### General findings on effectiveness

For the Primary Sample, use of the micronutrients was associated with a 46% decrease in mean bipolar symptom severity scores at LOCF (Table 3), a change that was significant (*t*(119) = 8.5, *p *< .001, ES = 0.78). With respect to the ADHD symptoms, the mean decrease from baseline to LOCF was 40% and was also significant (*t*(28) = 3.3, *p *< .002, ES = 0.62).

**Table 3:** Symptom severity at Baseline and at Last Observation Carried Forward

		Bipolar					ADHD				
		
		symptom severity (range 0 to 48)					symptom severity (range 0 to 9)				
					
row #	Data Sample	Baseline	Last Observation Carried Forward	%change from Baseline	effect size	sample size: N =	Baseline	Last Observation Carried Forward	%change from Baseline	effect size	sample size: N =
**1**	**Primary Sample: Bipolar = sum of sub-samples #2 and #3**										
	Mean	17.8	9.6 ***	-46%	0.78	120	5.0	3.0 *	-40%	0.62	29
	*Median*	*17.4*	*7.1*	*-59%*			*5.0*	*3.0*	*-40%*		
	Std. Deviation	10.1	8.3				2.9	2.2			

	**Baseline-Median Split Sample: split by Baseline bipolar symptom severity**						**split by Baseline ADHD symptom severity**				
**1 Above**	Above Baseline Median:										
	Mean	25.1	12.9 ***	-49%	1.21	60	7.5	3.1 ***	-59%	1.72	14
	*Median*	*24.0*	*10.9*	*-54%*			*7.0*	*3.0*	*-57%*		
	Std. Deviation	6.1	9.4				1.1	2.7			
**1 Below**	Below Baseline Median:										
	Mean	9.1	6.4 ***	-30%	0.45	60	2.7	2.9	7%	-0.10	15
	*Median*	*8.7*	*5.6*	*-36%*			*3.0*	*3.0*	*0%*		
	Std. Deviation	4.6	5.4				1.9	1.8			

**1 Older**	Age 12 or above:										
	Mean	17.1	9.3 ***	-46%	0.81	70	4.4	2.2 **	-50%	0.67	15
	*Median*	*16.0*	*7.1*	*-55%*			*4.0*	*1.4*	*-65%*		
	Std. Deviation	10.5	8.2				2.4	2.1			

**1 Younger**	Age younger than 12:										
	Mean	17.1	10.2 ***	-41%	0.73	50	5.7	3.8 *	-33%	0.54	14
	*Median*	*17.6*	*6.9*	*-61%*			*7.0*	*3.4*	*-51%*		
	Std. Deviation	8.6	8.5				3.3	2.2			

**1 Male**	Male: Mean	17.4	10.3 ***	-41%	0.67	71	4.9	3.0 **	-39%	0.59	21
	*Male: Median*	*17.0*	*7.2*	*-58%*			*5.0*	*3.0*	*-40%*		
	Std. Deviation	8.9	8.8				3.1	2.1			

**1 Female**	Female: Mean	18.3	8.7 ***	-53%	0.94	49	5.4	3.1	-43%	0.64	8
	*Female: Median*	*17.8*	*6.2*	*-65%*			*6.7*	*2.5*	*-63%*		
	Std. Deviation	9.3	7.5				2.5	2.7			

**2**	**Sub-Sample: Bipolar but not ADHD**										
	Mean	16.7	9.4 ***	-44%	0.80	91					
	*Median*	*15.7*	*6.6*	*-58%*							
	Std. Deviation	9.9	8.3								

**3**	**Sub-Sample: Bipolar and ADHD**										
	Mean	18.2	10.3 ***	-43%	0.72	29	5.0	3.0 **	-40%	0.62	29
	*Median*	*20.0*	*8.6*	*-57%*			*5.0*	*3.0*	*-40%*		
	Std. Deviation	9.0	8.3				2.9	2.2			

**4**	**Alternative Sample: ADHD but not bipolar**										
	Mean						6.0	3.2 ***	-47%	1.04	41
	*Median*						*6.7*	*2.8*	*-58%*		
	Std. Deviation						2.5	2.6			

Notes: *** indicates that mean LOCF is significantly different from mean at Baseline, at level p < .001; ** at level p < .01; *at level p < .05

There were several outliers with high bipolar symptom severity, making examination of the median scores more informative than mean scores (cf. Table 3). The median bipolar symptom severity was 59% lower at LOCF; the median ADHD symptom severity was 40% lower at LOCF.

Table 3 also reports on two sub-samples of the Primary Sample and an Alternative Sample. For those who reported having PBD but not ADHD (sub-sample in row 2), we found a 44% mean decrease in bipolar symptom severity (*t*(90) = 7.7, *p *< .001, ES = 0.8). For those with both ADHD and PBD (sub-sample in row 3), the mean decrease in bipolar symptoms was 43% (*t*(28) = 3.9, *p *< .001, ES = 0.72), and the mean decrease in ADHD symptoms was 40% (*t*(28) = 2.9, *p *< .002, ES = .62). Finally, for those with only ADHD but not bipolar, the mean decrease in ADHD symptoms was 47% (*t*(40) = 6.9(40), *p *< .001, ES = 1.04).

**Figure 2:**
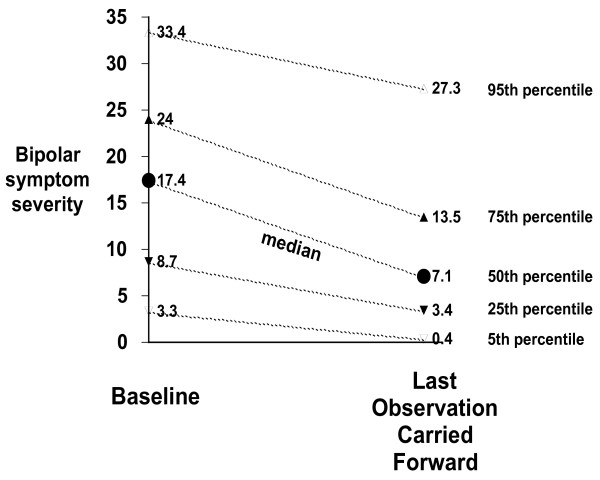
**Bipolar symptom severity at Baseline and at Last Observation Carried Forward: Median and Other Percentiles; Primary Sample**.

**Figure 3:**
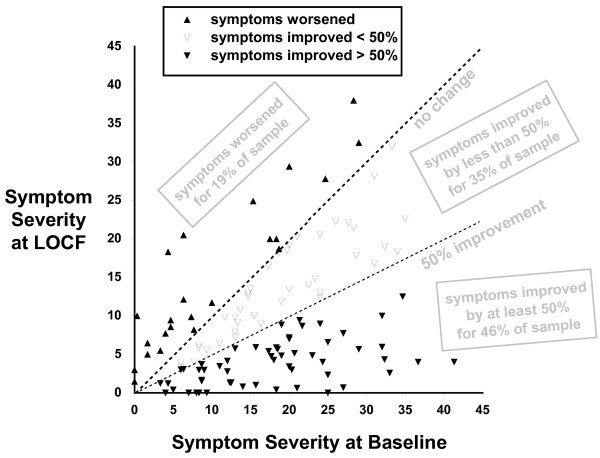
**Bipolar symptom severity at Baseline and Last Observation Carried Forward (LOCF), for all 120 clients in Primary Sample**.

Figure 2 shows bipolar symptom severity at baseline and at LOCF in terms of percentiles. However, this figure should not suggest that all clients experienced uniform reductions in symptom severity, or that the reductions were proportional to baseline symptom severity. There is substantial heterogeneity across clients in their symptom severity at baseline and in their response to the micronutrient treatment over time. In Figure 3, symptom severity at baseline is plotted against symptom severity at LOCF for all 120 clients. Most data points lie below the line indicative of no change, illustrating the finding that most clients experienced a reduction of symptom severity. At the LOCF, 46% of the sample experienced at least 50% improvement in symptoms and of these, 5% of the sample was symptom-free. Thirty-five percent of the sample experienced less than a 50% improvement in symptoms and 19% reported increases in symptom severity. Of the 19% of clients who worsened over time (23 of 120), many had low baseline scores to begin with (10 of 23 had baseline scores below 5), and only a few (7 of 23) experienced symptom worsening greater than 5 units.

In the ADHD literature, a 30% decrease in symptom scores is accepted as a good indicator of a clinically significant symptom reduction [44]. In this dataset, 55% of those with ADHD in the Primary Sample and 76% of the Alternative Sample (ADHD but not bipolar) showed a 30% reduction in ADHD symptom severity. In contrast, the bipolar disorder literature typically uses a 50% reduction as a clinically meaningful change: for the Primary Sample 46% showed at least a 50% reduction in bipolar symptom severity. Similar measures for the various samples and sub-samples can be found in Table 4.

**Table 4:** Percent of clients who experienced a minimum % reduction in symptom severity from Baseline to Last Observation Carried Forward

	Bipolar symptom severity: % reductions from Baseline		ADHD symptom severity: % reductions from Baseline	
	
	> 30%	> 50%	> 30%	> 50%
1. Primary Sample: Bipolar = sum of sub-samples #2 and #3 (N = 120)	65%	46%	55%	45%
				
2. Sub-sample: Bipolar but not ADHD (N = 91)	68%	45%		
				
3. Sub-sample: Bipolar and ADHD (N = 29)	55%	48%	55%	45%
				
4. Alternative Sample: ADHD but not Bipolar (N = 41)			76%	63%

We also analyzed the effect of age within the Primary Sample (Table 3). Using age 12 or above at baseline as the cut off for adolescents, we found significant declines from baseline to LOCF in bipolar symptoms were observed for both older (*t*(69) = 6.8, *p *< .001, ES = 0.81) and younger children (*t*(49) = 5.2, *p *< .001, ES = 0.73). We also found significant declines from baseline to LOCF in ADHD symptoms for both older (*t*(14) = 2.6, *p *< .01, ES = 0.67) and younger children (*t*(13) = 2.0, *p *< .03, ES = 0.54). Using two-sample t-tests for means, there were no statistically significant differences (at *p *< 0.05) between older and younger children at baseline or LOCF in either bipolar or ADHD symptoms.

The results for gender in the Primary Sample were similar (Table 3): statistically significant declines from baseline to LOCF in bipolar symptoms were observed for both males (*t*(70) = 5.7, *p *< .001, ES = 0.67) and females (*t*(48) = 6.6, *p *< .001, ES = 0.94). A significant decline from baseline to LOCF was also observed in ADHD symptoms for males (*t*(20) = 2.7, *p *< .01, ES = 0.59) but not females (*t*(7) = 1.8, *ns*, ES = 0.64). Using two-sample t-tests for means, there were no statistically significant differences (at *p *< 0.05) between males and females at baseline or LOCF in either bipolar or ADHD symptoms.

### Medication index

**Figure 4:**
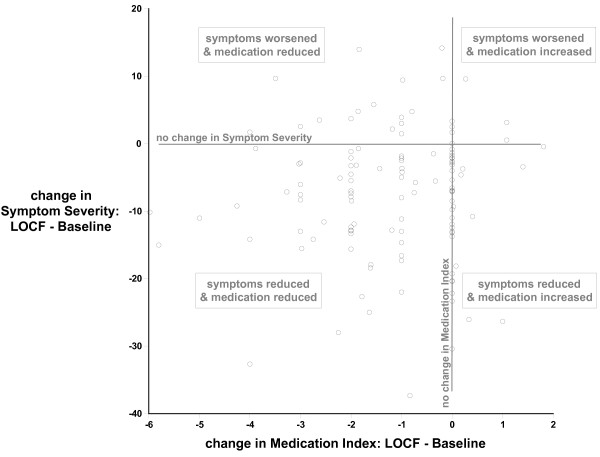
**The relationship between change in medication index and change in symptom severity from Baseline to Last Observation Carried Forward (LOCF)**.

As indicated in Table 2, medication indices decreased substantially from baseline to LOCF for both samples. Not only did the percent of children taking medication change but also the quantity of medication being consumed, as indicated by the changes in medication index. For example, in the Primary Sample, at baseline 79% of the sample were taking medications, but at LOCF, only 38% were using medications, a decrease of 52%. Further, for those taking medications at baseline, the amount of medication being taken dropped by 74%. Inspection of the data showed that majority of those clients who reduced their medications (60 of 76) did show symptom improvement. This relationship between medication change and symptoms is illustrated in Figure 4.

### Consideration of drop-outs

In order to assess whether those who stopped reporting earlier did so because of a deterioration in symptoms or lack of response, we also evaluated whether those who stopped earlier did so because they did not experience significant symptom improvement. Although we required that all clients in the sample reported for at least 60 days out of 180 days, there was variation in the last month that was reported. About half the clients (n = 59) reported through 6 months, while the remaining were distributed about evenly across the other months: 22 stopped reporting in month 3, 18 in month 4, and 19 in month 5. For the entire Primary Sample (n = 120), the median percent reduction in bipolar symptom severity from Baseline to LOCF was 46%; by last month reported, the median percent reduction was 23% for month 3, 55% for month 4, 50% for month 5, and 60% when month 6 was the last month reported. For each of these four groups of clients, there was a statistically significant reduction from Baseline symptom severity to LOCF (*p *< .001). We also compared those who stopped at 3, 4 and 5 months with those who continued to 6 months and found no meaningful differences. There was also no consistent relationship between continuation of symptom reporting and degree of reduction in symptom severity. Although those who stopped reporting at month 3 had higher mean symptom severity than those who continued submitting reports beyond month 3, the reverse was true for those who stopped in month 4 (and in month 5): those continuing to report had higher mean symptom severity than those who stopped.

## Discussion

These database analyses of 120 children with pediatric bipolar disorder and an alternative sample of 41 children with just ADHD symptoms revealed significant amelioration in symptoms for up to 6 months of observation. All families purchased a broad-based 36-ingredient micronutrient product and chose to track their child's progress on a mood checklist and, for some, an ADHD checklist. The data presented here were from people who submitted those checklists on at least 60 of the 180 days. The symptom decrease for the entire Primary Sample was about 46% if based on mean values; however, the presence of some outliers with very high scores makes mean changes less informative. Based on medians, symptom amelioration exceeded 59%. Another way to look at the results is in terms of individual responder status: 46% experienced >50% improvement at 6 months. The decrease in symptom severity was robust enough to be significant across gender, age (pre-adolescent versus adolescent clients) and presence or absence of ADHD.

Although these response rates are similar to those reported in the pharmaceutical literature on PBD, direct comparisons are limited due to the self-selection bias inherent in this design and the fact that response rates are typically higher in open label trials as compared with those reported in RCTs. The results are consistent with other published studies using this same micronutrient formula with adults with bipolar disorder [31] and adults with both mood dysregulation and ADHD [34].

These positive responses were achieved after significant reduction of psychiatric medications for many of the clients, which is a critical issue to families of children with emotional problems. The potential advantage of this method of intervention lies in the negligible side effects reported by clients [26,33] and the safety record of the product when monitored through biochemistry, blood pressure, weight and haematology [26,27,34,36]. Given the reluctance of many psychiatrists to prescribe medications in this younger age group and the concerns over side effects, if substantiated with more rigorously controlled trials, this micronutrient approach may be a viable and valuable alternative.

The relationship between medication use and symptom severity is difficult to disentangle in a database analysis. However, given the high rate of medication use at baseline, in combination with elevated scores on a rating scale assessing bipolar symptoms, it is clear that many of the children in this database were not experiencing full symptom relief from medications alone. With the addition of micronutrients, clients were able to reduce medication use and, for most of them, experience further improvement in their symptoms. There was a small subset of the sample who did worsen over time alongside a drop in medications, indicating that micronutrients do not, of course, assist all patients with psychiatric symptoms. This cross tapering is deliberate and is typically done in conjunction with the prescribing physician. There have been a few reports as far back as 1992 indicating that nutrients can amplify the effects of psychiatric medications. In that year, Bell and her colleagues reported that a mixture of B vitamins increased the efficacy of tricyclic antidepressants in 14 adults suffering from depression [43]. In another example, Popper [28] reported this phenomenon specifically with the micronutrient product evaluated here. Recently, the Harvard Mental Health Letter reported on four natural ingredients that boost the effect of psychiatric medications [45]. This interaction between micronutrients and psychiatric medication is generally received as good news by patients who would like to reduce their medication dosage.

The response rate of the subsample of those with both ADHD and PBD symptoms is of particular relevance given the overall lack of response of this subgroup to conventional mood stabilizing medications, estimated between 20-29% [13,22]. While it is important not to over interpret these data given the limitations associated with the design, we did observe a similar reduction in symptoms in those clients who had both ADHD and PBD (48% experienced >50% reduction in PBD symptoms by LOCF) as compared with a sample of clients with PBD but not ADHD (45% experienced >50% reduction in PBD symptoms by LOCF), at the very least suggesting that ADHD does not appear to alter the rate of improvement in bipolar symptoms.

Further, although it appears that the ADHD symptoms in those with both ADHD and PBD did not improve as much as those with ADHD-only (55% versus 76% if one takes 30% reduction as a good indicator of improvement in ADHD symptoms [44]), the ADHD-only sample showed a higher baseline value; the LOCF values were comparable. Although there was no statistical evidence of differences between these two groups, the effect size was larger for those with only ADHD in comparison with those with both ADHD and PBD (1.04 versus .62 respectively). These improvements in ADHD symptoms are consistent with those documented from an open label trial using this same formula in adults with ADHD and mood instability [34] but lower than trials using psychostimulants in the treatment of ADHD symptoms in children with ADHD and PBD [46].

Many researchers have speculated about the role micronutrients may play in moderating psychiatric symptoms. Some argue that symptom improvement is unlikely due to any one ingredient; minerals, vitamins and amino acids are critical to the synthesis of neurotransmitters and often are required *in combination *for optimal benefit; single nutrient approaches may not be sufficient to correct all imbalances due to the array of nutrients required for effective neurochemical synthesis [47]. Ames et al. [48] demonstrated that genetic diseases can reduce the binding affinity of enzymes, which in turn lowers the rate of metabolic reactions. Micronutrients function as cofactors in enzymatic reactions responsible for synthesizing and metabolizing neurotransmitters. It may be that only a broad-based micronutrient formula can correct and stabilize multiple functions, particularly in cases that have been resistant to other forms of treatment. Kaplan et al. [25] speculate that some forms of mental dysfunction may be caused by in-born errors of metabolism in key neurobiological pathways, in particular those responsible for neurochemical synthesis, second messenger signaling and uptake of neurotransmitters. Recent studies suggest that the manufacture of adenosine triphosphate (ATP), the energy source of the mitochondria, is compromised in bipolar disorder, ADHD and other mental disorders [49]-[50].

There are many limitations associated with a database analysis such as this one. Foremost, all of the data were based on parent report, and there was no corroborating clinician or teacher report. However, by its very nature, clinician report is based on patient-based information. Further, parents have been found to be as sensitive to monitoring change as teachers [51]. What would be useful for future studies would be to verify whether other functional changes occur, such as improved quality of life or improved neurocognitive functioning. These factors would provide more objective data to assess change as well as allow for further speculation about changes occurring at a neural level.

The clients were clinical patients and, as in any naturalistic study, were not assessed using structured interviews to confirm diagnosis. Even in research settings that use structured interviews, differentiating bipolar from ADHD is fraught with controversy in the pediatric literature and the diagnosis of PBD can be unreliable [2], with some researchers suggesting that mood instability should be deemed a core feature of ADHD [52]. The fact that most of the children were being treated using medications typical for these conditions gives us some confidence that they were experiencing symptoms to a level of impairment warranting the use of psychiatric medications; however, which diagnosis they would actually meet criteria for is impossible to establish from this database. Indeed, the low rate of ADHD diagnoses in the PBD sample (24%) as compared with the wider literature on the overlap does question the validity of the self-report. Research suggests that there is a low agreement between diagnoses made by clinical evaluation and diagnoses made by standardized diagnostic interviews [53], supporting the need for caution in generalizing these results to the extant pediatric bipolar population. Nevertheless, given the significant amount of controversy that surrounds PBD and its diagnosis, there is currently a lack of consensus on the diagnostic criteria [4] and as such, low validity is a problem common to the PBD literature as a whole. However, the rating scale used in this research monitored classic bipolar disorder symptoms as defined in the DSM and as such, our results reflect change in these traditional symptoms.

On a related note, the self-report questionnaire used to assess change in ADHD symptoms was not ideal. There were only three questions we could confidently analyse to monitor change in ADHD symptoms which makes it difficult to compare these results to those of other treatment studies for ADHD carried out in research environments. However, those three questions are the cardinal questions related to the ADHD diagnosis - assessing the three main problems associated with ADHD. Other research has confirmed that those three questions are reliable and valid indicators of the disorder [54].

Given the sample size, we could not address the impact that co-occurring conditions had on the response to the micronutrients. We are aware that many of the children reported other problems, such as anxiety, behavioural problems, and pervasive development disorders, likely making them more impaired than individuals typically recruited for pharmacological trials; however, we did not have enough information to independently assess the impact of co-occurring diagnoses on treatment response. Further, given that it was a database analysis, there are many other variables we cannot control for such as use of other nonpharmacological treatments such as psychotherapy, change in diet and nutritional status, and use of other nutritional products, such as omega-3 s or amino acid supplements, that may have been used in conjunction with these micronutrients. Compliance could only be assessed via client report (which is inherently problematic) and could not be confirmed via blood work or other more careful monitoring systems. Further, the large number of pills that the children are required to consume could be viewed as a disadvantage for many and may have contributed to the early drop outs. However, the formula is now available in a powder form, which may reduce the problems associated with consuming a large number of pills.

There are three types of self-selection bias inherent to this type of study: participation, reporting, and dropping out. With respect to participation bias, people searching for nutritional methods to manage their mood symptoms are likely to have been ineffectively treated by pharmaceuticals, and are not necessarily representative of everyone with bipolar disorder. In other words, many of the participants in this database could be considered treatment resistant, making these positive findings even more important clinically. Reporting bias relates to the fact that many people who purchase this product choose not to monitor their symptoms due to the time and effort involved in doing so as well as the impact of the illness itself; the possible extent of this bias cannot be analyzed with the available data, but most clients who purchased this product chose not to monitor symptoms consistently enough to be included in the analysis. Reporting bias could also result in more favorable reports, given that the clients chose the treatment that they received. Research shows that when participants choose their therapy, they are more likely to rate it as effective [55], thereby inflating the results reported and reducing the generalizability of the results found. However, the fact that a subset of the clients did not benefit over time or worsened (19% of the Primary Sample) reduces the likelihood that the results were entirely driven by a positive expectancy bias.

It is also possible that rather than choosing not to report on symptoms while consuming the formula, that these clients represent treatment failures; i.e., their lack of reporting was due to early discontinuation of the treatment, because they either found it ineffective, did not like the side effects or could not comply with the regimen, similar to reasons for drop-outs in RCT studies. Indeed, 709 children were found in the database but only 120 met our inclusion criteria for continued reporting, so we do not know what happened to the remaining 83% of the clients and to what extent their response rate mirrored those in this sample of 120. While it is tempting to draw parallels to well controlled designs and conclude that the treatment was not effective for these other clients, we simply cannot establish why reporting did not continue, in contrast to RCTs, where researchers know that a participant who stops reporting has also stopped taking the treatment because the investigators control the distribution of pills. As most of the children who were excluded from the Primary Sample ceased reporting within the first two weeks, any lack of change at that point would not have been due to lack of effectiveness given that it typically takes at least that amount of time before any effect is noted. Nevertheless, given that many who start taking this formula choose not to report on symptoms, we cannot establish with any certainly whether the response rate noted in the Primary Sample can generalize to the wider clinical population.

The third type of self-selection bias occurs as a result of non-responders ceasing to continue to report symptoms due to lack of improvement. About 50% of the Primary Sample stopped reporting before 6 months. Analysis of these "drop-outs" indicated that they did experience significant reduction in their symptoms, suggesting that they likely stopped reporting, not because of a lack of response, but because they found it tiresome to continue to monitor and report symptoms. A similar finding was reported in the adult sample [31].

The fact that we continued to see benefit through 6 months of reporting is a strength in that it reduces the likelihood that placebo, natural cycles of PBD, or positive expectancy effects were contributing to the positive changes observed. Having a control group would have given greater confidence that the changes observed were due to the consumption of the formula and not natural remission of the symptoms over time.

It is important to note that none of the authors was involved in any way with the data collection. Further, none of the authors and none of their Universities are commercially affiliated with the developer/manufacturer. Unlike drug trials, these clients were not being paid to complete questionnaires; indeed, they were paying to purchase the product.

## Conclusions

There are many methodological problems inherent in a database study, the most pertinent ones being its open label nature which naturally inflates the effectiveness of the treatment, and the low number of clients who opted to monitor their symptoms for any length of time. However, the results reported here, in combination with the other publications to date, illustrate a consistent pattern of improvements in psychiatric symptoms, providing a powerful case for further research on micronutrient treatment, in particular investment in RCTs. Although the current study cannot be generalized to population response rates and as such we urge caution to not over-interpret these results, a more carefully diagnosed sample of children exhibited similar reductions in psychiatric symptoms [26]. Funding such research is a challenge because unlike pharmaceuticals, there is no patent protection of micronutrients that would make the research a worthwhile investment. Despite the obstacles in both funding and also publishing this type of work [56], the consistently positive reported outcomes to date in the general absence of adverse side effects indicate that controlled clinical trials are essential next steps to further our understanding of the effect of micronutrients on mental health.

## Competing interests

The authors declare that they have no competing interests.

## Authors' contributions

All authors: 1) have made substantial contributions to conception and design, or acquisition of data, or analysis and interpretation of data; 2) have been involved in drafting the manuscript or revising it critically for important intellectual content; and 3) have given final approval of the version to be published.

## Pre-publication history

The pre-publication history for this paper can be accessed here:

http://www.biomedcentral.com/1471-244X/10/74/prepub

## Supplementary Material

Additional file 1 **Table S1**. EMPowerplus Capsule Ingredient List Click here for file

Additional file 2 **Table S2**. Symptom Rating Scale for Bipolar and ADHD symptoms Click here for file
