# Supplementary material for: Database analysis of children and adolescents with Bipolar Disorder consuming a micronutrient formula
Source: BMC Psychiatry. 2010 Sep 28;10:74. doi: 10.1186/1471-244X-10-74 (PMC2954995; doi:10.1186/1471-244X-10-74)
Supplement: Additional file 1 — Table S1. EMPowerplus Capsule Ingredient List [file 1471-244X-10-74-S1.DOC]

Table S1: EMPowerplus Capsule Ingredient List

1 cap 15 caps

Vitamin A 384.0 IU 5760 IU

Vitamin C 40.0 mg 600 mg

Vitamin D 96.0 IU 1440 IU

Vitamin E 24.0 IU 360 IU

Vitamin B1 1.2 mg 18 mg

Vitamin B2 0.9 mg 13.5 mg

Vitamin B3 6.0 mg 90 mg

Vitamin B5 1.4 mg 21.6 mg

Vitamin B6 2.4 mg 36 mg

Vitamin B9 96.0 ug 1440 ug

Vitamin B12 60.0 ug 900 ug

Vitamin H 72.0 ug 1080 ug

Calcium 88.0 mg 1320 mg

Iron 0.9 mg 13.74 mg

Phosphorus 56.0 mg 840 mg

Iodine 13.6 ug 204 ug

Magnesium 40.0 mg 600 mg

Zinc 3.2 mg 48 mg

Selenium 13.6 ug 204 ug

Copper 0.5 mg 7.2 mg

Manganese 0.6 mg 9.6 mg

Chromium 41.6 ug 624 ug

Molybdenum 9.6 ug 144 ug

Potassium 16.0 mg 240 mg

Proprietary Blend: Choline bitartrate, DL-phenylalanine, citrus bioflavonoids, inositol, Lglutamine, L-methionine, grape seed extract, ginkgo biloba (leaf), germanium

sesquioxide, boron, vanadium, nickel
