# Supplementary material for: Database analysis of children and adolescents with Bipolar Disorder consuming a micronutrient formula
Source: BMC Psychiatry. 2010 Sep 28;10:74. doi: 10.1186/1471-244X-10-74 (PMC2954995; doi:10.1186/1471-244X-10-74)
Supplement: Additional file 2 — Table S2. Symptom Rating Scale for Bipolar and ADHD symptoms [file 1471-244X-10-74-S2.DOC]

Table S2. Symptom Rating Scale for Bipolar and ADHD symptoms

**Rating Scale: Bipolar Symptoms**

1. Feeling worthless, helpless or hopeless
2. Sleeping more or less than usual
3. Eating more or less than usual
4. Hard to concentrate or decide
5. Loss of interest in hobbies or activities
6. Avoiding other people
7. Overwhelming feeling of sadness
8. Loss of energy, feeling very tired
9. Thoughts of death or suicide
10. An excessively high or elated mood
11. Unreasonable optimism or poor judgement
12. Hyperactivity or racing thoughts
13. Talkativeness, rapid speech, incoherent
14. Irritability
15. Extremely short attention span
16. Rapid shifts to rage or sadness

**Rating Scale: ADHD Symptoms**

1. Moves around a lot, fidgets
2. Impulsive
3. Short attention span, distractible
4. Demanding, can't be satisfied
5. Gets frustrated easily, explosive temper
6. Mood swings
7. Irritable, excitable
8. Cries easily
